# 

**Published:** 1906-12

**Authors:** 


THE AMERICAN

### JOURNAL OF DENTAL SCIENCE

*THE OFFICIAL ORGAN OF THE FLORIDA, GEORGIA AND SOUTH CAROLINA STATE DENTAL SOCIETIES.*

THIRD SERIES

VOL. XXXVII    DECEMBER, 1906    NO. 12

EDITORS

WM. GIRD BEECROFT, D. D. S., Madison, Wis.    B. H. TEAGUE, D. D. S., Aiken, S. C.

Published on the 10th day of each month.
Subscription price, $1.00 per year. Foreign countries, $1.30 per year.
*Entered as second-class matter, Madison, Wis.*

Address all communications, both business and editorial, to The Dental Publishing Company, Madison, Wis.

### BOOK NOTICE.

A System of Dental Surgery, by the late Sir John Tomes, F. R. S. Fifth edition. Revised and enlarged by Charles S. Tomes, M. A., F. R. S., and Walter S. Nowell, M. A., Oxon, P. Blakiston's Sons & Co., Philadelphia, Pa. Price, $4.50.

A quarter of a century ago no work on dental surgery, not excepting that of Chapin Harris, was conceded more thorough as a text book than the first editions of Tomes. According to the English standard they were exhaustive in technical and scientific description and arrangement of the subject matter, as well as complete in detail of practice. The authors have carefully revised and enlarged the work, bringing it up to date in all lines of practice. But it is in the treatment of dental lesions that the work is most efficient in detail and appeals most acceptably to the real dental surgeon. Reference to its pages will aid in many instances of complicated cases. No progressive practitioner should be without it.

**Twenty-Fifth Anniversary Reunion, Celebration and Clinic of the Chicago College of Dental Surgery Alumni Association.**

On the sixteenth and seventeenth days of January, 1907, the Alumni Association of the Chicago College of Dental Surgery will celebrate the twenty-fifth anniversary of the establishment of the college by holding a grand reunion and clinic. Arrangements have been made for a number of papers, a very extensive clinic, a theater party, and a banquet. A railroad rate of a fare and a third for the round trip from all points in the United States and Canada on the certificate plan has been arranged for.

A cordial invitation is extended to the general profession to be present, as well as all members of the Alumni Association and all graduates of the college.

R. C. Brophy,
J. P. Buckley,
Committee on Publicity.

